# Assaying Homodimers of NF-κB in Live Single Cells

**DOI:** 10.3389/fimmu.2019.02609

**Published:** 2019-11-07

**Authors:** Erik W. Martin, Sayantan Chakraborty, Diego M. Presman, Francesco Tomassoni Ardori, Kyu-Seon Oh, Mary Kaileh, Lino Tessarollo, Myong-Hee Sung

**Affiliations:** ^1^Laboratory of Molecular Biology and Immunology, National Institute on Aging, National Institutes of Health, Baltimore, MD, United States; ^2^Laboratory of Receptor Biology and Gene Expression, National Cancer Institute, National Institutes of Health, Bethesda, MD, United States; ^3^Mouse Cancer Genetics Program, National Cancer Institute, National Institutes of Health, Frederick, MD, United States

**Keywords:** RelA, NF-κB, transcription factor, number and brightness, microscopy, oligomerization, dimerization

## Abstract

NF-κB is a family of heterodimers and homodimers which are generated from subunits encoded by five genes. The predominant classical dimer RelA:p50 is presumed to operate as “NF-κB” in many contexts. However, there are several other dimer species which exist and may even be more functionally relevant in specific cell types. Accurate characterization of stimulus-specific and tissue-specific dimer repertoires is fundamentally important for understanding the downstream gene regulation by NF-κB proteins. *In vitro* assays such as immunoprecipitation have been widely used to analyze subunit composition, but these methods do not provide information about dimerization status within the natural intracellular environment of intact live cells. Here we apply a live single cell microscopy technique termed Number and Brightness to examine dimers translocating to the nucleus in fibroblasts after pro-inflammatory stimulation. This quantitative assay suggests that RelA:RelA homodimers are more prevalent than might be expected. We also found that the relative proportion of RelA:RelA homodimers can be perturbed by small molecule inhibitors known to disrupt the NF-κB pathway. Our findings show that Number and Brightness is a useful method for investigating NF-κB dimer species in live cells. This approach may help identify the relevant targets in pathophysiological contexts where the dimer specificity of NF-κB intervention is desired.

## Introduction

**Figure 1:**
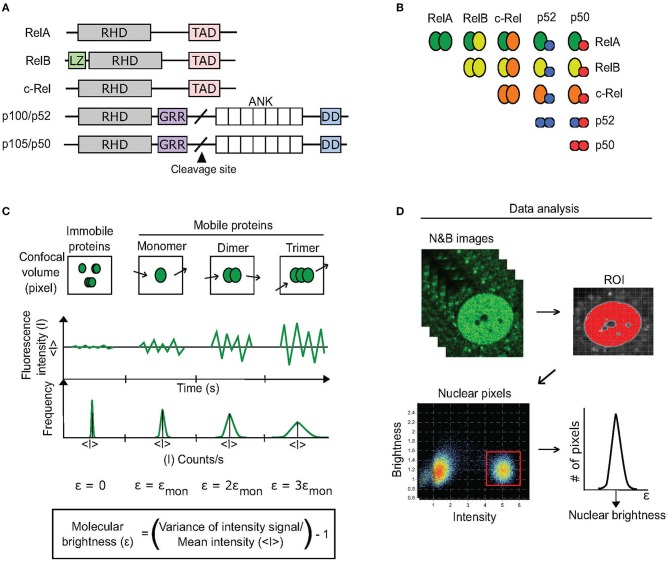
N&B approach to discern dimerization status of RelA. **(A)** There are five homologous proteins of the NF-κB TF family. All contain a Rel homology domain (RHD) that promotes dimerization with other RHD-containing proteins as well as DNA binding. RelA, RelB, and c-Rel each also contain a transcription-activation domain (TAD) that enables them to activate transcription. Cleavage of p105 and p100 produces p50 and p52, respectively. Additional domains include: LZ, leucine zipper; GRR, glycine-rich region; ANK, ankyrin-repeat domain; DD, death domain. **(B)** 15 potential NF-κB dimers exist based on RHD interactions. **(C)** The N&B assay measures the oligomer state of a protein by determining its molecular brightness (ε) within a region of interest (ROI) in a cell. A fluorescent protein's molecular brightness is determined by calculating the fluctuations (variance) in mean fluorescence intensity (<I>) that are caused by the movement of protein oligomers (monomers, dimers, trimers, k-mers) within every pixel (confocal volume) of the ROI over time (confocal imaging acquisition). The ratio of the variance to the mean fluorescence intensity of the pixels is equal to the protein's brightness (ε) + 1. Because immobile proteins do not produce such movement-based fluctuations, their molecular brightness is equal to 0. **(D)** For quantifying a protein's brightness within an ROI, in our case the nucleus, the brightness values of each pixel comprising the ROI are extracted from a stack of N&B images (see Methods). The brightness values are then fitted to a Gaussian distribution to determine the protein's overall brightness within the ROI.

Nuclear Factor-kappaB (NF-κB) is arguably the most important signaling pathway involved in immune responses (1). The specificity of NF-κB action as a transcription factor (TF) is partly mediated by the particular dimers that translocate into the nucleus in response to extracellular stimuli or stress (Figure 1A). In the nucleus, NF-κB homo- and hetero-dimers (Figure 1B) reversibly interact with specific DNA sequence motifs to activate the transcription of hundreds of target genes (2, 3). Depending on which of the 5 different NF-κB TF proteins comprise the dimers that translocate to the nucleus (4), different gene expression profiles can be induced (5, 6). Yet, because the NF-κB TF family is comprised of up to 15 different dimer species (1) (Figure 1B), developing a thorough understanding of how individual NF-κB dimers regulate transcription has proved to be an exceedingly difficult task.

Molecular biology approaches such as immunoprecipitation, immunoblotting, and EMSAs are not particularly well-suited for investigating more than a few dimer species simultaneously. Importantly, they cannot be used to analyze dimers in live intact single cells and do not distinguish between different oligomer species. Advances in imaging technologies have revolutionized biological research by superseding many of the constraints inherent to classical molecular biology techniques. In addition to enabling the visualization of molecular biological processes in real-time, microscopy techniques enable high throughput time-course measurements from the same individual cell in a relatively direct and non-invasive manner. Despite the importance of NF-κB, to our knowledge only a few imaging studies have investigated the dimerization status of this TF in living cells, using fluorescence cross-correlation spectroscopy (FCCS) or Förster-resonance energy transfer (FRET) (7–9).

The Number and Brightness (N&B) assay is a live-cell imaging technique that measures the aggregation or oligomerization state of proteins of interest in a specific area of a cell, such as the nucleus (10). Its use has revealed insights into the oligomerization status of crucial TFs, including the hormone receptor transcription factors: glucocorticoid receptor (GR) (11–13), androgen receptor (AR) (12), and progesterone receptor (PR) (12); as well as CCAAT/enhancer-binding protein alpha (C/EBPα) (12). It has also been used to quantify the aggregation of DNA (14), Huntingtin (15), and amyloid peptides (16), as well as other proteins (17–26). Besides a brief inclusion in a report (13), the N&B assay has not been used to study NF-κB TF dimers.

We conducted a series of experiments to explore the technological feasibility of using the N&B assay to measure the homodimer status of the NF-κB TF RelA in single living cells. We discovered that the N&B assay detects the presence of a mixed RelA dimer status in the nuclei of stimulated immortalized and primary fibroblasts, with RelA homodimers seemingly comprising a substantial proportion of the overall RelA dimer species. Moreover, we obtained evidence suggesting that the N&B assay can be used to quantify pharmacological perturbations of NF-κB dimers.

## Methods

### Materials

The mEGFP-N1 (54767) plasmid from Michael Davidson, and the RelA cFlag pcDNA3 (20012) (27) from Stephen Smale, were purchased from Addgene. The pSF-EF1α-Ub-Neo vector (OG606) was purchased from Oxford Genetics. The mEGFP-mutGR (monomeric glucocorticoid receptor (GR) mutant; monomer control) (GFP-GRN525), mEGFP-GR (wild-type GR), mEGFP-AR (androgen receptor), and mEGFP-PR (progesterone receptor) plasmids, as well as the dihydrotestosterone (DHT) and progesterone (PR), were previously described (12) and kindly provided by the Hager lab (NIH, Bethesda, MD). Additional reagents included lipopolysaccharide (LPS) (Enzo Life Sciences; ALX-581-008); TNFα (R&D; 410-MT-010); dexamethasone (Dex) (Sigma; D4902); withaferin A (WFA) (681535; EMD Millipore); and trichostatin A (TSA) (Sigma; T8552). Primary antibodies included polyclonal rabbit anti-RelA (Santa Cruz; SC-372), monoclonal rabbit anti-p50 (Santa Cruz; sc-114) (which also detects p105), polyclonal rabbit anti-RhoGDI (Sigma; R3025), and monoclonal rabbit anti-GAPDH (Cell Signaling Technology; 14C10). Secondary antibody consisted of polyclonal anti-rabbit IgG (Jackson Immunoresearch; 211-035-109).

### Cloning

To generate the mEGFP-RelA plasmid encoding an N-terminal fusion protein, cDNA was amplified by PCR using Phusion polymerase mix (New England BioLabs (NEB); M0532S) and the indicated primers (Table S1). Constructs were digested with restriction enzymes from NEB (EcoR1-HF, R3101S; EcoRV-HF, R3195S; BsrGI-HF, R3575S) and ligated into the pSF-EF1α-Ub-Neo vector using Promega LigaFast Rapid DNA Ligation kit (Fisher Scientific; PR-M8221). The resulting plasmid was used to transform DH5α competent cells (ThermoFisher; 18265017). Plasmid derived and expanded from a single antibiotic resistant clone was purified using an EndoFree Plasmid Maxi kit (Qiagen; 12362). Plasmid construction was verified by DNA sequencing.

### Cell Culture and Transfections

NIH3T3 cells (CRL-1658) were purchased from ATCC. Cells were cultured and maintained in a 37°C humidified environment of 5% CO_2_/95% air in growth media composed of phenol-red-free Dulbecco's Modified Eagle's Medium (DMEM) (Gibco; 21063-029) supplemented with 10% fetal bovine serum (FBS) (Gemini Bioproducts; 100-500) and 100 units/mL penicillin, 100 μg/mL streptomycin, and 2 mM L-glutamine (1% p/s/g) (Gibco; 15140-122). Passaging of cells was performed using brief exposure to 0.25% trypsin-EDTA (Gibco; 25200-056). Cells in DMEM containing only 10% FBS (lacking p/s/g) were transiently- or stably-transfected with the respective expression vectors using Fugene HD (Promega; E2311) according to the manufacturer's protocol. Pools of cells with stable-integration of EF1α-promoter driven mEGFP-RelA were obtained via selection in G418 sulfate solution (500 μg/mL) (Mirus; MIR5920). Expression and function of the fluorescent fusion proteins was tested through a combination of immunoblotting and confocal microscopy (Figure S1). Primary mouse adult fibroblasts (MAFs) for the N&B assay were obtained from ear pinna minced and digested in 200 μg/mL Liberase TM (Sigma; 5401119001) in a 37°C water bath for 1 h. Digested pinna were then diluted 5-fold in growth media and centrifuged at 1,000 rpm for 5 min. Liberase-containing media was aspirated, and cells were resuspended in growth media and cultured for ~1 week. Upon reaching greater than ~50%, but less than ~90% confluence, primary fibroblasts were passaged using 0.25% trypsin-EDTA using routine cell culture procedures. Primary cells were passaged no more than twice before being used in the study.

### Immunoblotting Lysates of Stably-Transfected Cells

Cells were lysed in ice-cold RIPA cell lysis buffer (Millipore; 20-188) containing Complete Ultra Mini protease inhibitors (Roche; 05892970001). Lysates were vortexed, centrifuged at 13,000 rpm for 15 min, and homogenized using insulin syringes. Concentrations of proteins were estimated using Protein Assay Dye Reagent Concentrate (Biorad; 5000006). Samples containing equal amounts of protein were heated at 95°C for 5 min in LDS sample buffer (ThermoFisher; NP0007) containing 10% 2-Mercaptoethanol (Gibco; 31350-010) and separated by SDS-polyacrylamide gel electrophoresis (PAGE) using 4–12% NuPage Bis-Tris pre-cast gels (ThermoFisher; NP0322BOX) and MOPS buffer (ThermoFisher; NP0001). Proteins were transferred using transfer buffer (ThermoFisher; NP00061) and 0.45 μm PVDF membranes (Millipore; IPVH00010). Membranes were blocked for 30 min in 5% (w/v) non-fat milk and then sequentially incubated with primary and HRP-conjugated secondary antibodies. HRP activity was detected using SuperSignal West Pico Chemiluminescent Substrate (ThermoFisher; 34580).

### Microscopy for Generation of Stably-Transfected Cells

All imaging was performed using a Zeiss LSM880 AxioObserver confocal microscope with an associated environmental chamber for live-cell imaging. During imaging all cells were maintained in a 37°C humidified environment of 5% CO_2_/95% air. Cells were seeded at medium confluence on 35 mm glass-bottomed dishes (MatTek; P35G-1.5-20-C) and cultured overnight before imaging in phenol-red-free growth media. Images were acquired using a 488 nm laser and a 40X/1.4 NA Plan-Apochromat oil-immersion objective with a fully open pinhole (600 μm).

### Microscopy for the N & B Assay

All imaging was performed using the same hardware and cell culture conditions as described above, unless described otherwise. Before imaging, cells were treated with indicated concentrations of ligands, as described in the Results section and associated figure legends. Treatment was not required to induce nuclear localization of the monomeric control (mEGFP-mutGR) as it is constitutively nuclear. mEGFP-GR nuclear localization was induced by pre-incubating cells with Dex (100 nM) for at least 1 h prior to imaging. mEGFP-GR maintained nuclear localization throughout the course of the experiments which typically lasted no more than 1–2 h after beginning imaging (per 35 mm dish). When mEGFP-RelA-expressing cells were treated with TNFα (10 ng/mL) or LPS (100 ng/mL), subsequent nuclear translocation was evident within 15–45 min and persisted for no more than ~1 h during which time N&B imaging was performed. For each nucleus analyzed, N&B microscopy was performed as previously described with minor adjustments (12). Briefly, a time-lapse stack of 150 images (256 × 256 pixels) was acquired using a 63X/1.4 NA Plan-Apochromat oil-immersion objective; a pinhole corresponding to 1 Airy unit; and a zoom of 6.6. Dwell-time per pixel was 8.24 μs with a subsequent frame scan time of 1.27 s. Laser power (488 nm) was set at 3% to detect near endogenous levels of mEGFP-RelA (Figure S1). Fluorescence signal was detected using a gallium arsenide phosphide (GaAsP) detector set to photon-counting mode. Additional imaging parameters include performing the 16-bit acquisition using a digital gain of 0.2. Images were acquired using one direction scanning, rather than bidirectional, to ensure a fixed re-sampling time of the mobile fluorescent proteins. No averaging was performed for line acquisitions. Nuclei were excluded from imaging if: they were not the only nuclei within a cell; they were radically misshapen or not intact; or if they had an approximate average fluorescence intensity under 4 or >17 (well-below pixel saturation values). Moreover, if nuclei exhibited significant rotational movement, or horizontal or lateral movement of greater than ~1 μm, they were discarded from analysis. The first 10 frames of each image stack were discarded to remove the initial impact of photobleaching from measurements. Image stacks were analyzed using the N&B option of the “GLOBALS for Images” software developed by the Laboratory for Fluorescence Dynamics (University of California, Irvine, CA), with the divider set to 1.

### Dimeric Population Estimation

Since N&B cannot separate mixtures of oligomeric states, the resulting brightness value represents a weighted-average combination of the species present in the illumination volume (10). In general, the dependence of the brightness value is given by a non-linear combination of the brightness and the occupation number of each species (28). Assuming RelA can only exist in monomeric or dimeric forms, then the expected brightness (ε_exp_) obtained by the N&B assay is given by:

$$\begin{array}{l} \varepsilon_{exp}=\frac{\varepsilon mon^{2}\;*\;Nmon+\varepsilon dim^{2}\;*\;Ndim}{\varepsilon mon\;*\;Nmon+\varepsilon dim\;*\;Ndim} \end{array}$$

Where εmon and εdim represent the brightness of the monomeric and dimeric species (i.e., values of 1 and 2, respectively); and Nmon and Ndim are the molar fraction of monomers and dimers, respectively.

### Mice

The *Nf*κ*b1*^−/−^*Rel*^−/−^ mice (herein referred to as p50/c-Rel double-KO mice) were generated by intercrossing *Nf*κ*b1*^−/^^−^ mice (B6.Cg-*Nf*κ*b1*^*tm*1*Bal*^/J; Jackson Laboratory) and *Rel*^−/−^ mice (29). The mEGFP-RelA knock-in (KI) mice were generated by inserting the mEGFP coding sequence (without stop codon) after the start codon (ATG) of RelA at the endogenous locus using CRISPR/Cas9 editing technology. Briefly, specific sgRNAs targeting the proximity region of RelA start codon were designed using the online tool MIT CRISPR Design (crispr.mit.edu) and generated *in vitro* using MEGAshortscript T7 transcription kit (Thermo Fisher Scientific; AM1354). sgRNAs were purified using MEGAclear kit (Thermo Fisher Scientific; AM1908). A double strand (ds) DNA donor template (~7 kb) containing the mEGFP fusion sequence was obtained from Genewiz (genewiz.com). Cas9 mRNA (TriLink Biotechnologies; L-6125), sgRNAs and dsDNA donor template were microinjected into one-cell stage zygotes obtained from C57BL/6Ncr × B6D2F1/J mice to generate mEGFP-RelA KI animals (EM, FTA, LT, MHS, in preparation). *Nf*κ*b1*^−/^^−^*Rel*^−/−^ and mEGFP-RelA KI MAFs were obtained as described above. Wild-type equivalent *Nf*κ*b1*^+/+^*Rel*^+/+^ MAFs were obtained from B6.129-Il12b^tm1Lky^/J mice (Jackson Laboratory), as the p50/c-Rel double-KO mice were bred using mice homozygous for both alleles. All MAFs were obtained from female mice aged 7–14 weeks. All mice were maintained under specific pathogen-free conditions at the animal facility of National Institute on Aging, and animal care was conducted in accordance with the guidelines of NIH.

### Co-immunoprecipitation of RelA Dimers

Whole-cell extracts from 10 × 10^6^ cells per condition were prepared in 1 mL of cell lysis buffer containing 1% NP-40, 50 mM Tris-HCl (pH 7.5), 150 mM NaCl, and Complete Ultra Mini protease inhibitors (Roche; 05892970001). Lysates were centrifuged at 13,000 rpm for 10 min at 4°C. The cleared supernatants were collected and 50 μL of the supernatant was saved as the input sample. For immunoprecipitation, 1.5 μg of rabbit anti-RelA antibody (Santa Cruz; sc-372) was added to 80 μL of Dynabeads protein A (Invitrogen; 10001D) in 500 μL of phosphate-buffered saline (PBS) containing 0.1% Tween-20 (PBST) for 20 min at room temperature to form bead-antibody complexes. The remaining cell lysate supernatants (~950 μL) were added to the bead-antibody complexes and incubated with rotation overnight at 4°C. The beads were washed three times with PBST and then the immunoprecipitated complexes were eluted in 25 μL of a 1:1 dilution of elution buffer (Invitrogen; 10006D) and 2xSDS sample buffer (Novex; LC2676) by heating at 70°C for 10 min. 25 μL of eluted protein complexes were resolved with a 8–16% Tris-Glycine gel (Invitrogen; XP08160) and visualized by immunoblotting using indicated antibodies.

### Statistical Analysis

Quantitative data are represented as median values with their respective quartiles unless stated otherwise. Significance (relative to controls) was tested using unpaired two-tailed Student's *t* test. *p*-values ≤ 0.05 were deemed statistically significant.

## Results

### RelA Homodimers Comprise a Substantial Proportion of Nuclear NF-κB in Live 3T3 Fibroblasts

Briefly, the N&B assay measures the oligomer state of a protein (monomer, dimer, multimer) within a cell by determining its molecular brightness (ε) via confocal microscopy (10) (Figures 1C,D). To do so, the approach relies on fluorescently-tagged proteins and specialized image analysis software (see Methods). In a model system, the molecular brightness of fluorescently-tagged proteins that exist as monomers is set to one. The fluorescent proteins that form homodimers have molecular brightness value of two, while k-mer proteins have brightness of k (Figure 1C). In cells, proteins can also exhibit intermediate values of molecular brightness (e.g., ε = 1.5-fold the brightness of monomers), which can be indicative of mixed populations of oligomeric species.

**Figure 2:**
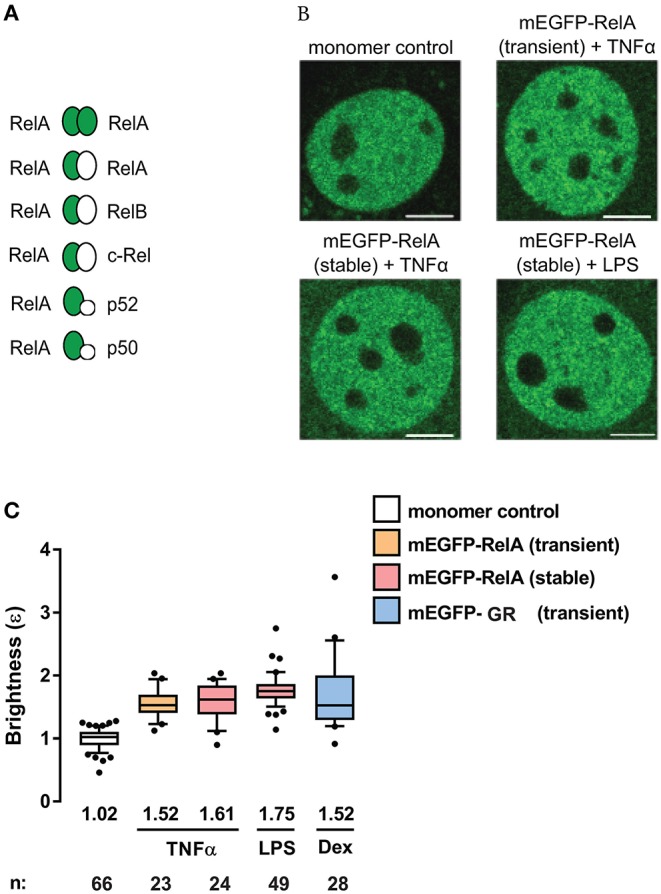
RelA exhibits substantial homodimer levels in mouse fibroblasts. **(A)** Schematic of possible mEGFP-RelA (green ellipse) interactions with mEGFP-RelA or other non-mEGFP-tagged NF-κB proteins (white ellipse). **(B)** Representative confocal micrographs of nuclei in 3T3 fibroblasts transiently-expressing monomer control, transiently-expressing mEGFP-RelA, or stably-expressing mEGFP-RelA under different treatment conditions. Image intensity scale was adjusted for optimal viewing. Scale bar: 5 μm. **(C)** Quantification of nuclear mEGFP-RelA brightness (ε) values relative to the monomer control in transfected cells (B) treated with 10 ng/ml TNFα, 100 ng/ml LPS, or 100 nM Dex. Data was obtained from at least two independent experiments performed on different days. Whiskers are drawn down to the 10th percentile and up to the 90th percentile. Number of nuclei and median values of each sample are presented below each boxplot.

To investigate the state of RelA-containing dimers using the N&B assay, we transiently-transfected NIH3T3 (3T3) fibroblasts with a plasmid encoding the fluorescent fusion protein mEGFP-RelA. We also generated 3T3 cells stably-expressing mEGFP-RelA (Figure S1). The cells were then treated with one of two stimuli [tumor necrosis factor-alpha (TNFα), which produces a quick but oscillatory response (30), or lipopolysaccharide (LPS), which produces a delayed response (31, 32)]. Relative to a mEGFP-tagged monomer control with a brightness of ~1, we observed a median brightness (ε) value of 1.52 for RelA in the nucleus of transiently-transfected cells treated with TNFα (Figures 2A–C). Because of the numerous possible RelA-containing dimers among which only mEGFP-RelA-tagged homodimers are presumed to produce brightness values of ε = 2, while the others are to give ε = 1 (Figure 2A), the observed brightness value suggests that RelA homodimers form a considerable portion (roughly 35%, see Methods for details) of all potential RelA dimer species in individual fibroblasts (Figure 2A). When 3T3 fibroblasts stably-expressing mEGFP-RelA were treated with TNFα, a similar nuclear brightness value of 1.61 (~43% dimers) was detected (Figures 2B,C). Furthermore, when treated with LPS, a nuclear RelA brightness value of 1.75 (~60% dimers) was obtained (Figures 2B,C). As a comparison control, we transiently-transfected 3T3 fibroblasts with cDNA encoding a different TF, mEGFP-tagged glucocorticoid receptor (mEGFP-GR), which is known to form homodimers in the nucleus (13). Upon treating mEGFP-GR-expressing cells with the GR-ligand dexamethasone (Dex), we observed a nuclear brightness value of ε = 1.52 (Figures 2B,C), suggesting that a substantial portion of nuclear GR (including dimers containing endogenous untagged GR) was present as homodimers of mEGFP-GR, as expected.

To confirm whether the N&B assay, in our hands, could quantify the presence of TF oligomers with brightness values greater than those of RelA or GR, we assayed TFs known to form higher-order oligomers in the nucleus. Fibroblasts were transiently-transfected with mEGFP-AR (androgen receptor) or mEGFP-PR (progesterone receptor) and treated with their ligands dihydrotestosterone or progesterone, respectively (Figure S2). Upon activation, we observed a nuclear brightness value of ε = 2.67 for AR and a brightness of ε = 2.07 for PR, indicating that AR and PR form higher-order oligomers in immortalized fibroblasts (Figure S2) as previously shown (12). Moreover, detection of brightness values ε > 2 verified a sufficiently wide N&B dynamic range for studying various oligomers using this assay. As we did not perform the experiments in RelA, GR, AR, or PR knockout cells, we could not quantify the impact of untagged endogenous RelA, GR, AR, and PR proteins on the observed brightness data.

### Primary Fibroblasts Maintain RelA Homodimers Independently of c-Rel and p50

**Figure 3:**
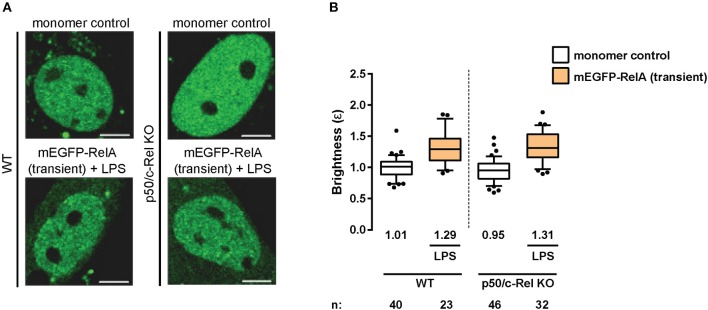
RelA homodimers in primary fibroblasts. **(A)** Representative confocal micrographs of nuclei in wild-type (WT) and p50/c-Rel double-KO primary MAFs transiently-expressing monomer control or mEGFP-RelA. The latter were treated with LPS (100 ng/mL). Image intensity scale was adjusted for optimal viewing. Scale bar: 5 μm. **(B)** Nuclear RelA brightness (ε) values relative to the monomer control in transfected primary fibroblasts **(A)** treated with 100 ng/ml LPS. Data was obtained from at least two independent experiments performed on different days. Whiskers are drawn down to the 10th percentile and up to the 90th. Number of nuclei and median values of each sample are presented below each boxplot.

Having observed RelA homodimers representing a substantial proportion of RelA-containing dimers in immortalized fibroblasts, we next sought to extend our analysis to primary cells. Therefore, we transiently-transfected wild-type (WT) mouse adult fibroblasts (MAFs) with plasmids encoding the monomer control or mEGFP-RelA, and treated the RelA-transfected cells with LPS. The N&B assay resulted in a relative RelA nuclear brightness value of ε = 1.29 in the primary fibroblasts (Figures 3A,B). While this value indicates a relatively lower proportion of RelA homodimers in comparison to immortalized 3T3 fibroblasts, it confirms that a substantial portion (roughly 20%) of RelA-containing dimers in the nucleus exist as homodimers in stimulated primary fibroblasts.

As previous studies have indicated the presence of RelA:p50 and RelA:c-Rel heterodimers (33), we next tested whether eliminating RelA binding partners (p105/p50 and c-Rel) would result in an increased relative abundance of RelA homodimers. To that end, we obtained MAFs from p50 (*Nf*κ*b1*)/c-Rel (*Rel*) double-knock-out (KO) mice and performed the N&B assay. Surprisingly, we obtained a nuclear RelA brightness value of ε = 1.31 in the double-KO cells (Figures 3A,B) relative to the monomer control, suggesting roughly equivalent levels of RelA homodimers in the WT and double-KO fibroblasts. The result was likely due to tagged RelA monomers readily forming dimers with untagged RelA monomers made available due to the lack of c-Rel and p50 protein (Figure 2A), as well as interactions with other untagged NF-κB TF monomers (Figure 2A), rather than substantially increasing levels of tagged RelA homodimers.

### Dimerization of NF-κB Subunits Can Be Perturbed by Small Molecules

**Figure 4:**
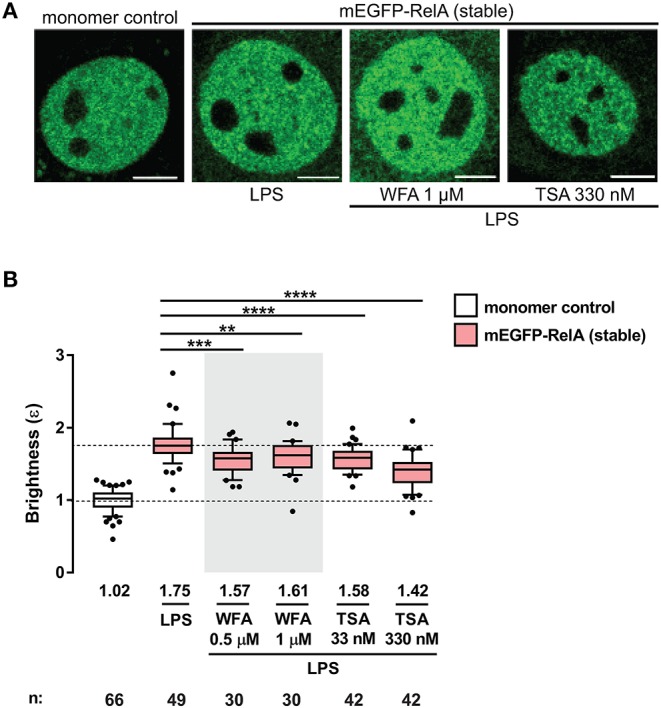
Perturbation of RelA homodimers by pharmacological agents. **(A)** Representative confocal micrographs of nuclei in transiently- and stably-transfected 3T3 fibroblasts pre-incubated with WFA or TSA before LPS treatment (100 ng/mL). Control and LPS images are the same as those in Figure 2B. Image intensity scale was adjusted for optimal viewing. Scale bar: 5 μm. **(B)** Brightness (ε) values of nuclear RelA in fibroblasts under different conditions. Control and LPS samples are the same as those in Figure 2C. Data was obtained from at least two independent experiments performed on different days. Whiskers are drawn down to the 10th percentile and up to the 90th. Number of nuclei and median values of each sample are presented below each boxplot. Unpaired two-tailed Student's *t*-test (**p ≤ 0.01, ***p ≤ 0.001, and ****p ≤ 0.0001) was performed for statistical comparisons.

A recent study by Dikstein and colleagues reported that Withaferin A (WFA), a naturally occurring anti-inflammatory and anti-cancer phytochemical, disrupts RelA dimerization by interacting with a conserved hydrophobic core domain and dimerization scaffold within RelA and other NF-κB subunits (34). To investigate whether small molecules such as WFA can disrupt RelA-containing dimers in living cells, we performed the N&B assay on the 3T3 fibroblasts stably-expressing mEGFP-RelA, pretreated with either 0.5 or 1 μM WFA for 1 h and stimulated with LPS (Figure 4A). Due to the pleiotropic effects of WFA, including IKKβ hyperphosphorylation (35), WFA concentrations >1 μM completely inhibited nuclear translocation of mEGFP-RelA. Therefore, we used lower concentrations ( ≤ 1 μM) for our assay. RelA nuclear brightness values decreased from ε = 1.75 for LPS-only treated cells (Figure 4B) to ε = 1.57 or ε = 1.61 for cells exposed to either 0.5 μM or 1 μM WFA prior to LPS treatment, respectively (Figure 4B). Although modest, the WFA-induced decrease in the brightness of RelA suggests that WFA perturbs the abundance of RelA homodimers and that such changes are quantifiable using the N&B assay.

We also examined the effects of another small molecule, trichostatin A (TSA), a class I and II histone deacetylase (HDAC) inhibitor which alters the global chromatin landscape by increasing the level of acetylated histones. To test whether RelA dimerization is affected by TSA, 3T3 fibroblasts stably-expressing mEGFP-RelA were pretreated with TSA for 2 h before LPS stimulation and N&B analysis (Figure 4). Interestingly, TSA-pretreatment significantly reduced brightness values of RelA. RelA brightness ε decreased from 1.75 in the nuclei of LPS-only treated cells (same control as for WFA) to 1.58 or 1.42 in the nuclei of TSA-pretreated cells, depending on the concentration of TSA (Figure 4B). Since the fraction of chromatin-bound TFs can range from 20% to 50% in live cells (36, 37), an intriguing possibility is that the global alteration in chromatin induced by TSA may influence the dimerization status of RelA. Such a reverse (gene to TF) action has been reported, where specific chromatin interfaces result in allosteric conformational changes of GR which impact gene-specific regulation (38). On the other hand, the HDAC inhibitor effect may be partly through non-histone targets, including acetylation of RelA (39–41) or RelA-regulating proteins (42, 43). The mechanisms of TSA action underlying the unexpected dimer perturbation are beyond the scope of this report and will require separation of effects on histones and non-histone targets.

Finally, we attempted to measure the perturbation of RelA-containing complexes (RelA:RelA, RelA:p50, and RelA:p105) by WFA and TSA using the conventional methods of immunoprecipitation and immunoblotting. While a trend toward a reduced abundance of RelA:p105 was observed for LPS-treated cells pre-incubated with WFA or TSA, the overall results were inconclusive (Figure S3). We suspect that the *in vitro* biochemical methods lack the sensitivity for detecting subtle changes in TF dimer composition that occur within intact live cells, highlighting the potential utility of the N&B assay.

## Discussion

The function of NF-κB TFs has been widely studied over the years in various cell-types and biological contexts. However, studies focusing on the dimerization states of NF-κB TFs have been relatively scarce due to the difficulties associated with obtaining and interpreting *in vitro* biochemical data. Nevertheless, using systems-based *in silico* modeling and experimental validations, a previous study indicated that RelA homodimers constitute ~25% of total RelA-containing dimers in mouse embryonic fibroblasts (44). The remainder of RelA dimers were determined to be comprised of other dimers, mostly RelA:p50 (44) (Figure 2A). Our N&B data from primary transfected MAFs suggest ~20% of RelA-containing dimers are RelA:RelA homodimers (Figures 3A,B), which is in accordance with the aforementioned study. However, whereas p105/p50 KO embryonic fibroblasts had increased abundance of endogenous RelA homodimers (to nearly 50% of the total RelA-dimer population) (44), our data suggest that ectopically expressed mEGFP-tagged RelA likely dimerizes with untagged RelA and other NF-κB subunits in the absence of p105/p50 and c-Rel in primary adult fibroblasts (Figures 3A,B).

A significant caveat of our study and many live-cell imaging approaches is the use of ectopically expressed fluorescent fusion proteins as well as the presence of untagged endogenous proteins. We mitigated the complications associated with ectopic expression by avoiding cells showing excessively high mEGFP signal; all our N&B data were from individual cells expressing low levels of the transgene. To obtain more definitive information about the composition of NF-κB dimers in living cells, it is imperative to study their biophysics in primary cells where the endogenous locus encoding the relevant subunit is replaced by its fluorescent fusion construct. Such a fluorescent knock-in (KI) reporter would retain the natural regulatory environment and would not harbor any untagged proteins (45). N&B assay of such KI reporter systems would enable accurate quantification of relative compositions of different NF-κB homodimers and heterodimers in real-time in single living cells. Toward this end, we recently generated such a KI mouse strain. When the N&B assay was performed on TNFα or LPS-treated MAFs obtained from the mEGFP-RelA KI mouse (in which the entire population of RelA protein was tagged with mEGFP (Figure S4) (see Methods), the results suggested again that RelA homodimers constitute a significant portion of the overall RelA dimer species in primary fibroblasts (slightly <35%).

The potential utility of the N&B assay in finding drugs that target NF-κB dimerization is evidenced by our observation that pre-treatment with withaferin A (WFA) or trichostatin A (TSA) modestly but significantly reduces RelA homodimer levels upon activation by LPS. Based on the primary roles that RelA and other NF-κB TFs fulfill in various immunological and pathological contexts, drug-induced perturbation of NF-κB dimers may have potent and clinically desirable consequences. With improvements in automated microscopy, the N&B assay may be useful in a drug screening platform in future high-throughput studies of NF-κB dimerization status. We also expect that the N&B assay and other live-cell imaging approaches will continue to reveal valuable information about NF-κB and other TFs in their natural states, thereby shedding light on the functional role of their biophysical characteristics.

## Data Availability Statement

The materials and data that support the findings of this study are available upon reasonable request.

## Ethics Statement

This study was carried out in accordance with the guidelines of the National Institute on Aging (NIA) and approved by the NIA Animal Care and Use Committee.

## Author Contributions

EM, SC, and M-HS designed the experiments and interpreted results. DP provided training, plasmids, and valuable assistance for the N&B assay. EM and SC performed the imaging experiments, data analysis, and drafted the manuscript. K-SO designed and performed the co-immunoprecipitation. MK crossbred the p105/p50 and c-Rel KO strains to produce the double KO strain. EM and FT generated the mEGFP-RelA knock-in mice. LT supervised the generation of the knock-in mice. M-HS revised the manuscript with input from all.

### Conflict of Interest

The authors declare that the research was conducted in the absence of any commercial or financial relationships that could be construed as a potential conflict of interest.
