# Supplementary material for: Assaying Homodimers of NF-κB in Live Single Cells
Source: Front Immunol. 2019 Nov 7;10:2609. doi: 10.3389/fimmu.2019.02609 (PMC6853996; doi:10.3389/fimmu.2019.02609)
Supplement: Supplementary file 1 [file Data_Sheet_1.pdf]

Supplementary Material

Assaying Homodimers of NF-κB in Live Single Cells

Erik W. Martin[1#], Sayantan Chakraborty[1#], Diego M. Presman[2&], Francesco Tomassoni Ardori[3], Kyu-Seon Oh[1], Mary Kaileh[1], Lino Tessarollo[3], Myong-Hee Sung[1]*

[1]Laboratory of Molecular Biology and Immunology, National Institute on Aging, National Institutes of Health, Baltimore, MD, USA.

[2]Laboratory of Receptor Biology and Gene Expression, National Cancer Institute, National Institutes of Health, Bethesda, MD, USA.

[&]Current address: IFIBYNE, UBA-CONICET, Universidad de Buenos Aires, Facultad de Ciencias Exactas y Naturales, Argentina.

[3]Mouse Cancer Genetics Program, National Cancer Institute, National Institutes of Health, Frederick, MD, USA.

[#] These authors contributed equally to this manuscript.

***Correspondence:** Myong-Hee Sung, sungm@mail.nih.gov

Running title

Probing dimerization of NF-κB

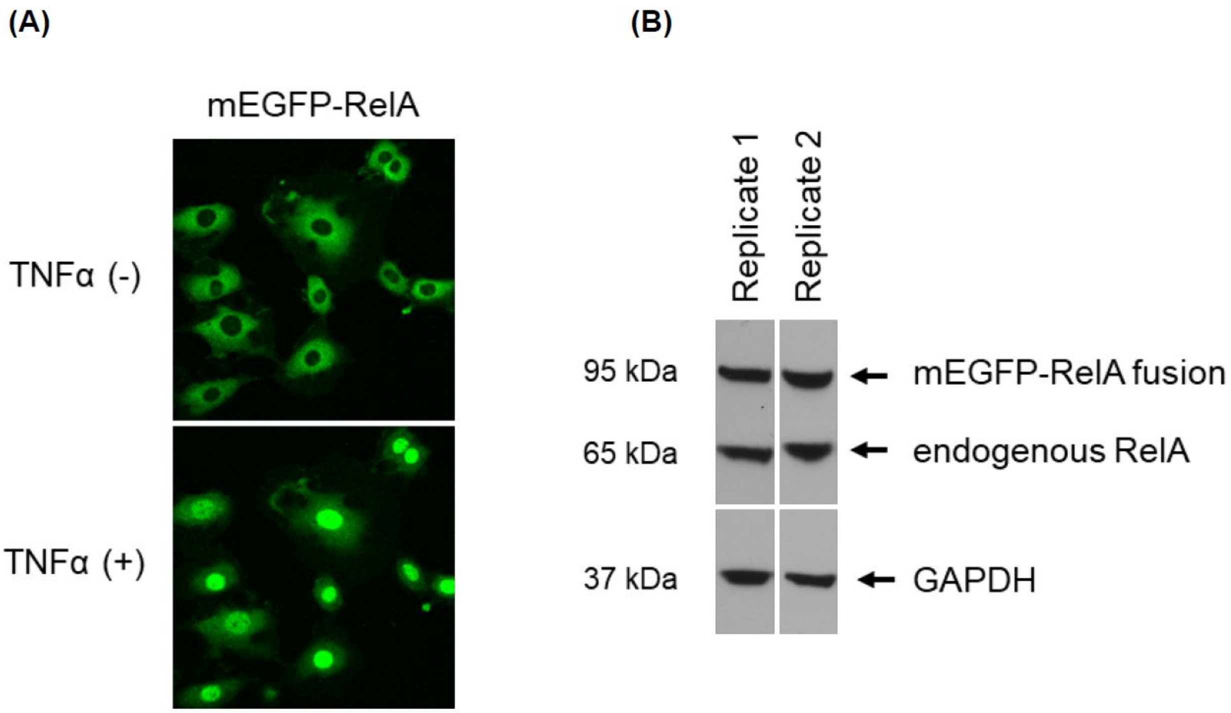

Figure S1. Expression and function of mEGFP-RelA protein. (**A**) Pools of mEGFP-RelA stably-transfected cells were treated with TNFα (10 ng/mL), or mock treated, respectively. Confocal microscopy was performed to image the expression and fluorescence of mEGFP-RelA, as well as its nuclear translocation following TNFα treatment. Image intensity scale was adjusted for optimal viewing. **(B)** The stably-transfected cells (without TNFα treatment) were lysed and immunoblotted using polyclonal antibodies to RelA to detect endogenous RelA and mEGFP-RelA proteins, as indicated in Methods. Experiments were repeated at least twice.

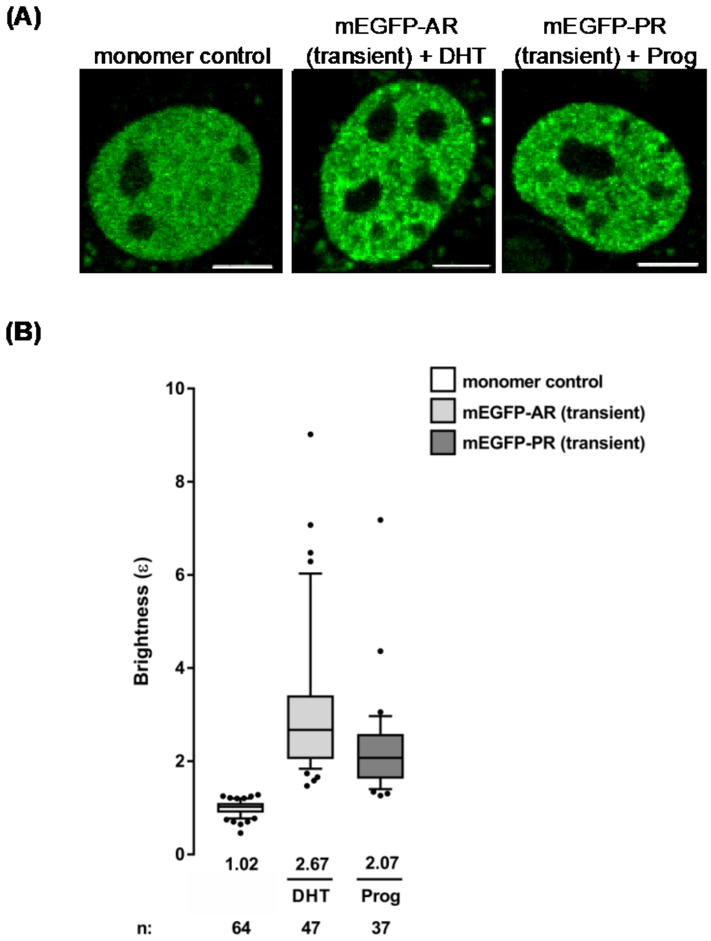

Figure S2. Androgen receptor (AR) and progesterone receptor (PR) form higher-order oligomers. **(A)** Representative confocal micrographs of nuclei in 3T3 fibroblasts transiently-expressing monomer control, mEGFP-AR or mEGFP-PR. Also indicated are different treatment conditions: 100 nM dihydrotestosterone (DHT) or 100 nM progesterone (Prog). The control image is the same as those in Figures 2B and 4A. Image intensity scale was adjusted for optimal viewing. Scale bar: 5 µm. **(B)** AR and PR brightness (ε) values in fibroblasts (A) under different conditions. Control samples are the same as those in Figures 2C and 4B. Data was obtained from at least two independent experiments performed on different days. Whiskers are drawn down to the 10th percentile and up to the 90th. Number of nuclei and median values of each sample are presented below each boxplot.

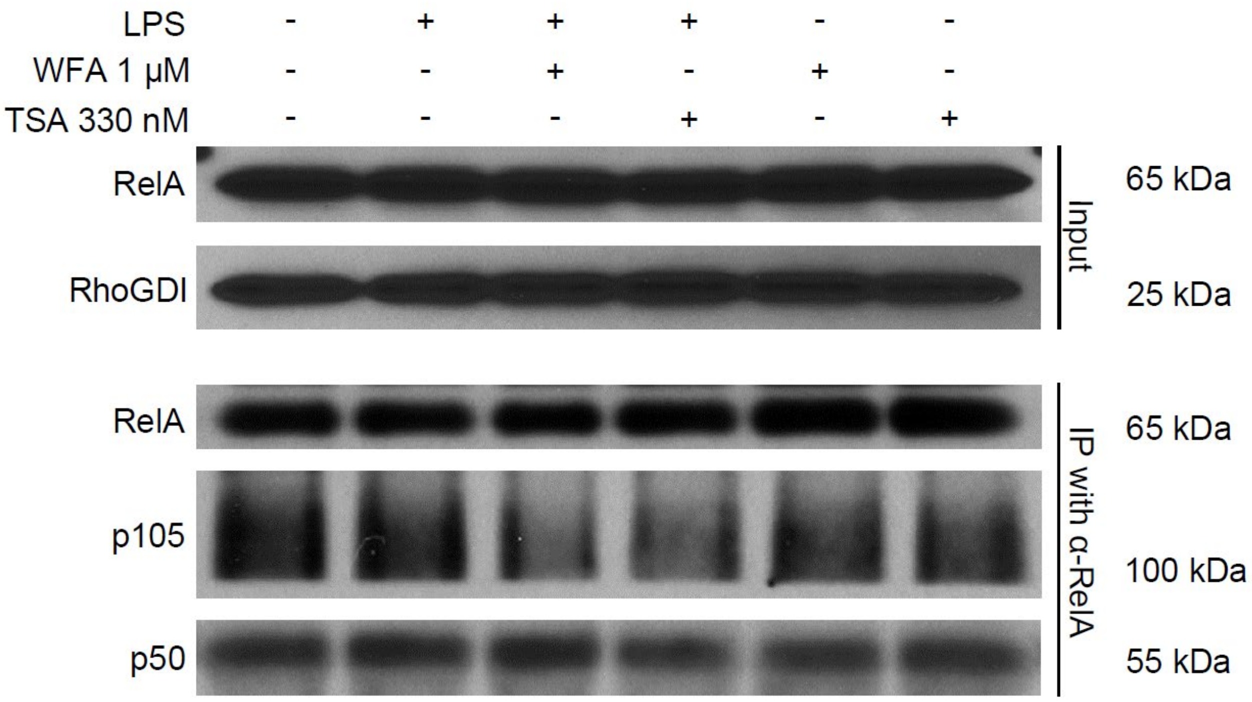

Figure S3. Immunoprecipitation and immunoblotting of RelA/p50 heterodimers. Co-IP of RelA and p105/p50 from 3T3 fibroblasts under indicated treatment conditions; LPS (100 ng/mL). Protein immunoprecipitated with anti-RelA antibody was probed for RelA and p105/p50 to determine changes to RelA dimer abundances under various conditions. Input consisted of whole cell lysates probed with anti-RelA and anti-RhoGDI antibodies, as indicated.

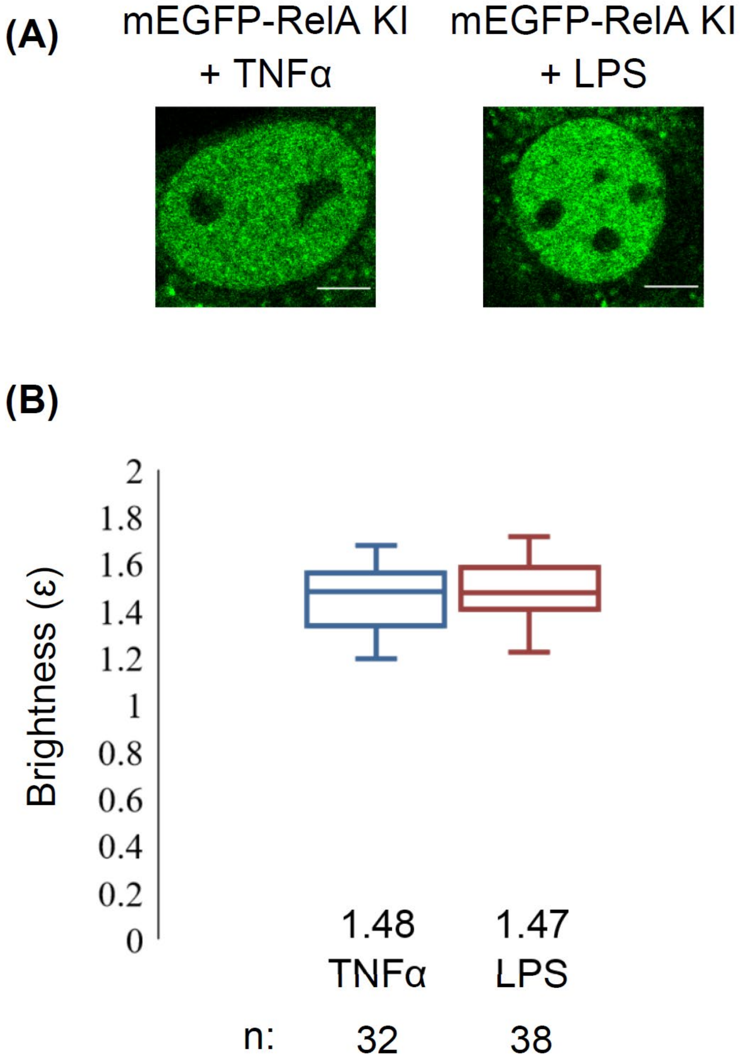

Figure S4. Detection of mEGFP-RelA homodimers in non-transfected primary fibroblasts. (A) Representative confocal micrographs of nuclei from MAFs obtained from mEGFP-RelA KI mice under different treatment conditions. Image intensity scale was adjusted for optimal viewing. Scale bar: 5 µm. **(B)** Quantification of nuclear mEGFP-RelA brightness (ε) values in MAFs treated with 10 ng/ml TNFα or 100 ng/mL LPS, relative to the monomer control. Monomer control samples are the same as those in Figure 3B (transfected WT control cells). Data was obtained from fibroblasts derived from at least four mice; ligand treatments were performed on separate days. Whiskers indicate the minimum and the maximum. Number of analyzed nuclei and median values of each sample are presented below each boxplot.

Forward mEGFP	ATATATGAATTC**GCCACC***ATG*GTGAGCAAGGGCGAGGA
Reverse mEGFP	ATATATTGTACAGCTCGTCCATGCCGA
Forward RelA	ATATATTGTACAAG*ATG*GACGATCTGTTTCCCCT
Reverse RelA	ATATATGATATC*TTA*GGAGCTGATCTGACTC

Table S1. Primers to generate the expression plasmid containing the mEGFP-RelA coding sequence. A leader sequence begins each primer. RE sites are underlined. The Kozak sequence is in bold. The start and stop codons are in italics.
